# Damage Localization in Composite Plates Using Wavelet Transform and 2-D Convolutional Neural Networks

**DOI:** 10.3390/s21175825

**Published:** 2021-08-30

**Authors:** Guillermo Azuara, Mariano Ruiz, Eduardo Barrera

**Affiliations:** Instrumentation and Applied Acoustics Research Group, Universidad Politécnica de Madrid, C/Nikola Tesla, s/n, 28031 Madrid, Spain; g.azuara@upm.es (G.A.); mariano.ruiz@upm.es (M.R.)

**Keywords:** ultrasonic guided waves, structural health monitoring, machine learning, convolutional neural networks, wavelet transform, damage imaging

## Abstract

Nondestructive evaluation of carbon fiber reinforced material structures has received special attention in the last decades. Usage of Ultrasonic Guided Waves (UGW), particularly Lamb waves, has become one of the most popular techniques for damage location, due to their sensitivity to defects, large range of inspection, and good propagation in several material types. However, extracting meaningful physical features from the response signals is challenging due to several factors, such as the multimodal nature of UGW, boundary conditions and the geometric shape of the structure, possible material anisotropies, and their environmental dependency. Neural networks (NN) are becoming a practical and accurate approach to analyzing the acquired data using data-driven methods. In this paper, a Convolutional-Neural-Network (CNN) is proposed to predict the distance-to-damage values from the signals corresponding to a transmitter-receiver path of transducers. The NN input is a 2D image (time-frequency) obtained as the Wavelet transform of the acquired experimental signals. The distances obtained with the NN are the input of a novel damage location algorithm which outputs a bidimensional image of the structure’s surface showing the estimated damage locations with a deviation of the actual position lower than 15 mm.

## 1. Introduction

The aeronautical industry’s interest in composite materials has increased significantly in the last decades, to the point that leading companies in the sector are currently manufacturing aircraft structures using composites accounting for more than 50% by weight [1,2]. The suitability of Carbon Fiber Reinforced Polymer (CFRP) materials for aerospace applications relies on their exceptional mechanical properties, such as a higher stiffness-to-weight ratio than metallic materials [3]. However, elevated production costs and issues related to critical damage, such as impact-induced delaminations, make it necessary to elaborate an advanced maintenance and repair methodology that monitors the structure’s health [4].

In recent years, Structural Health Monitoring (SHM) has become one of the most interesting techniques to evaluate aeronautical structure health, mainly when using Ultrasonic Guided Waves (UGW), such as Lamb waves, with surface-bonded piezoelectric transducers (PZT) [5,6,7]. Damage detection and localization using this technique have traditionally focused on knowledge of the material’s physical properties and the effective extraction of damage-sensitive features from the acquired signals. However, the application of SHM to composite materials presents several challenges related to their inherent anisotropies, elastic wave damping, and the dispersive nature of UGW.

Machine Learning (ML) methods [8] are used widely in other fields, such as image classification and speech recognition. They allow putting aside physical knowledge to perform purely data-driven methodologies. The advantage of ML methods and, particularly, solutions using Deep Learning (DL) (i.e., using multiple layers in the network) [9], relies on the ability to build models from sample data (i.e., the training dataset) to effectively make predictions without determining the previous relationships between input and output data. The Convolutional Neural Networks (CNN) approach [10] uses a class of deep neural networks based on an architecture implemented with convolutional filters (i.e., kernels) applied to the input signal or image.

The evaluation of structural systems with ML applications, such as wind turbines [11], automobiles [12], rotating machinery [13], and civil structures [14,15,16,17], has demonstrated their ability to perform reliable predictions. Recent advances in aerospace structure SHM using ML methods have demonstrated applications for impact location (e.g., using a 2-D CNN to detect the impact zone [18], a time-series signal rearranged as 2-D images for classifying both the structure’s status and the impact zone via CNN [19], and the impact coordinates using an Artificial Neural Network (ANN) [20]), damage localization in plate-like structures (using acceleration data [21] and simulating damage located in different locations with rearranged time-series signals to 2-D images [22], or using spectrograms by time-varying 1-D CNN [23]) and fatigue prognosis [24]. The previous solutions were based on both 1-D and 2-D CNNs, but computer vision-based CNN inputs are two-dimensional images, usually with three channels for RGB codification [10,25,26].

In this work, the acquired waveforms were preprocessed using a Morlet Wavelet Transform (MWT), converting the time-domain signal into a 2-D array including time-domain and frequency-domain information. Wavelet Transform (WT) has been widely used in time-frequency fault diagnosis [27] and vibration-based rolling bearing evaluation [28]. In addition, WT methods have been primarily used in Lamb wave-based SHM instruments due to their ability to extract meaningful features from 1-D signals, such as Time of Flight (ToF) [29,30], and frequency correlations [31,32,33].

In our research, a 2-D CNN was applied to estimate the normal distance from the direct transmission path to the damage using wavelet-transformed 1-D experimental UGW signals (particularly, the residual signals obtained from the responses to two different excitation levels), propagating through a composite plate as inputs. Two different approaches with CNNs were considered: a regression output and a classification output. The obtained distances were subsequently introduced into a novel damage location algorithm for obtaining a color-map image showing the estimated locations of the damage. The combination of 2-D CNNs, with their advanced capabilities to extract meaningful characteristics from a disperse dataset (with high variance in the input data, due to their dependence on several conditions, such as temperature and boundary conditions), and the novel algorithm designed for this study, presents a powerful tool for damage localization in composite plates.

Section 2 presents the physical background on which the feature extraction is based. Section 3 introduces the methodology followed in the paper for distance estimation (experimental setup, data acquisition process, data preprocessing, designed CNN models, and the formulation of the imaging algorithm). Section 4 shows the results from both the CNN training process and the imaging results. The final sections present the discussion, conclusions and future application of the study.

## 2. Physical Background

Elastic waves propagating through thin plates result in UGWs and, particularly, Lamb waves [34]. In a solid plate-like medium, these waves propagate, interacting with boundaries and defects, resulting in “wave packets” in a multimodal way [35]. UGWs are very powerful for SHM applications due to their large-range inspection, good wave-damage interaction, and cost-effectiveness.

PZT transducer distribution over a plate-like structure has been deeply studied throughout the last decades [36,37,38], resulting in several studies trying to solve the influence of the transducer distribution in the imaging algorithms [39,40,41,42]. The influence of damage on wave propagation may result in signal attenuation, phase shifting, and higher harmonics generation [43,44]. These variations in signals can be easily quantified by performing signal processing and statistical analysis in both time-domain and frequency-domain.

**Figure 1:**
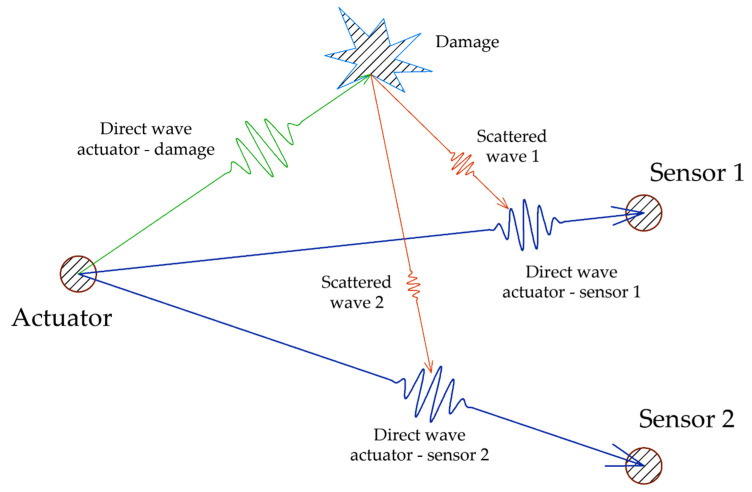
Scattering effects of damage outside the direct propagation path.

However, the influence of damage located far from the direct-transmission path, in a pitch-catch configuration, needs a deeper analysis of the acquired signals and it is usually laborious to obtain meaningful indicators. Figure 1 shows how the location of damage outside of two different direct-propagation paths can affect the guided wave.

**Figure 2:**
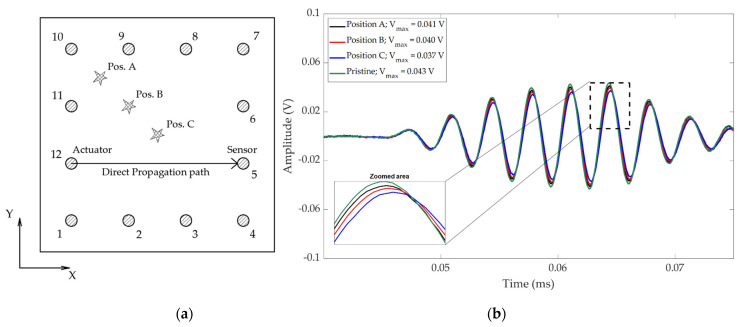
Scattering effects of three simulated damages outside the direct propagation path. The higher the distance from damage to the direct path, the lower the attenuation of the response signal. (**a**) Layout of the preliminary experiment. (**b**) Acquired signals propagated from the actuator to sensor (excitation signal was a 20 V amplitude, 3.5 cycles hanning-windowed, and 325 kHz toneburst).

These effects are depicted in Figure 2. Different simulated damage (using a putty-like Blu-Tack mass) were performed in a preliminary experiment carried out in a composite plate to analyze how the scattering effects influence the propagated direct wave. Using signal processing techniques, increases in amplitude and phase delay-shifting were observed as the path-to-damage distance decreased. However, the slight amplitude differences made it difficult to extract robust indicators from these signals using traditional signal processing.

## 3. Methodology

This paper presents the methodology followed to implement a 2-D CNN network to locate damage using experimental data from a composite-material plate. The following paragraphs detail the experimental setup, the data generation and acquisition process for network training, the preprocessing of the time-series signals to convert them into a two-dimensional array, the architecture of the proposed CNNs, and the imaging algorithm designed for this method.

### 3.1. Experimental Setup Configuration

**Figure 3:**
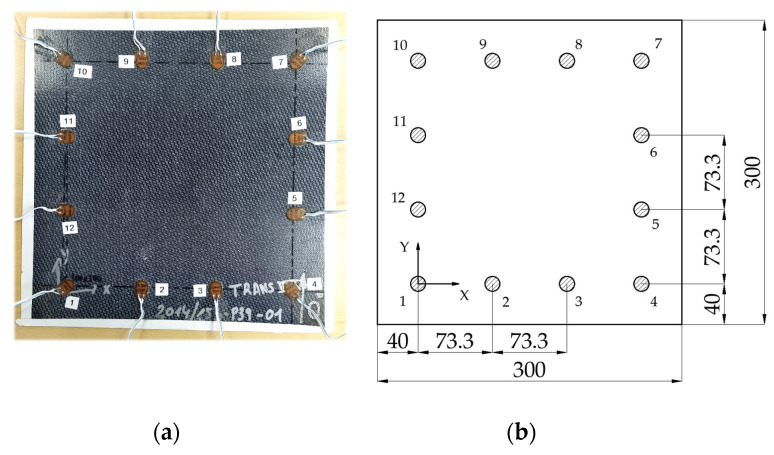
Tested specimen. (**a**) Real picture of the specimen with cables and transducers attached. (**b**) Detailed drawing with significant dimensions (in mm).

In this study, a thermoplastic matrix composite plate was used to propagate the UGW through it (Table 1). Twelve PiCeramic PZT transducers model DuraAct P-876K025 (with active disc D10 mm × TH 0.2 mm) were bonded to its surface using an epoxy adhesive from DELO (AD821). They were wired to the 12-channels of an embedded electronic system [45] capable of generating and acquiring electric signals with a sampling frequency of 12.5 MS/s. Figure 3 shows the specimen and the simplified drawing showing its dimensions.

### 3.2. Data Acquisition Process for Generating the Training Dataset

**Figure 4:**
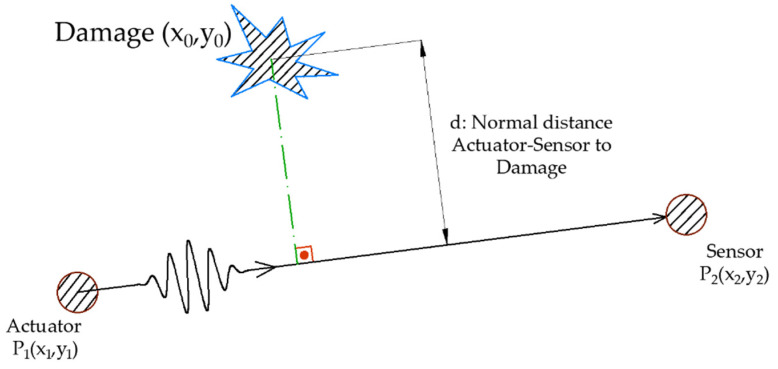
Sample labeling: normal distance from damage point to the direct propagation path.

DL-based applications require the generation of labeled data to perform training and create the relationship between input and output. As stated in the introductory section, the estimated magnitude is the normal distance from a direct transmission path to the damage. The formula used to compute the distance between a point and a straight line is Equation (1). The equation parameters are shown in Figure 4.

(1) $$d\left(P_{1}\left(x_{1},y_{1}\right),P_{2}\left(x_{2},y_{2}\right),D\left(x_{0},y_{0}\right)\right)=\frac{\left|\left(x_{2}-x_{1}\right)\left(y_{1}-y_{0}\right)-\left(x_{1}-x_{0}\right)\left(y_{2}-y_{1}\right)\right|}{\sqrt{{\left(x_{2}-x_{1}\right)}^{2}+{\left(y_{2}-y_{1}\right)}^{2}}},$$

**Figure 5:**
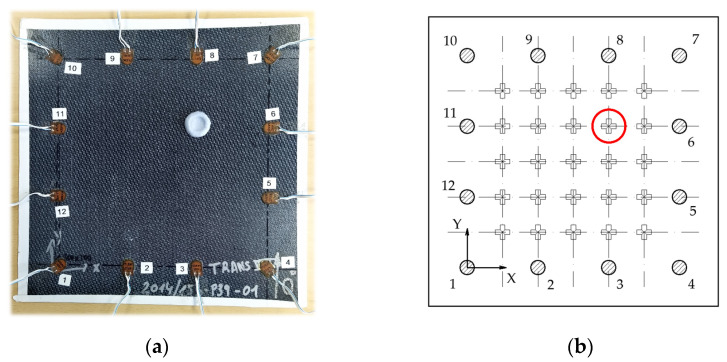
Training grid positions. (**a**) Specimen with attached Blu-Tack in the training grid position circled in (**b**). (**b**) The layout of the training grid, depicting the 25 training positions.

**Figure 6:**
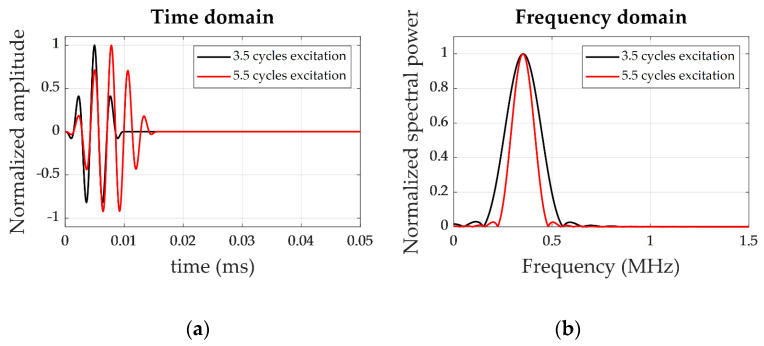
Example of 350-kHz excitation signals of 3.5 and 5.5 cycles. (**a**) Time-domain waveform. (**b**) Frequency domain.

A 5 × 5 positions grid was defined to obtain the measurements for the training dataset. The damage was simulated by attaching a 4-g, 2.5-cm diameter mass of Blu-Tack to the surface (see Figure 5). Blu-Tack was selected for simulating damage due to its mechanical properties, which cause both attenuation and phase shifting on UGW. Besides, the excitation conditions were varied by sweeping among different conditions: amplitude, frequency, and the number of cycles. All the excitation signals were hanning-windowed tonebursts, resulting in narrowband excitation (see Figure 6) with amplitudes of 10 and 20 V, frequency sweeping from 250 kHz to 350 kHz in 5-kHz steps, and the number of cycles from 3.5 to 6.5 in 0.5-cycles steps. The total number of different tests carried out was 7644 (2 different amplitudes × 21 frequencies × 7 cycles × 26 grid positions plus pristine). Since the pitch-catch configuration was selected, 12 transducers attached to the specimen resulted in 132 different signals per test (12 × 11, removing the pulse-echo signals), totalizing 1,009,008 (7644 × 132) waveforms.

### 3.3. Conversion of 1-D Waveforms to 2-D Arrays Using Wavelet Transform

**Figure 7:**
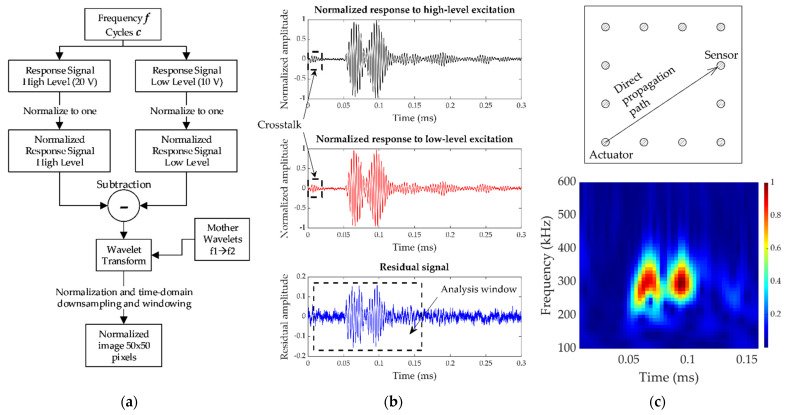
Training samples preparation. (**a**) Workflow for sample preparation. (**b**) Example of normalized high-level and low-level excitation responses and residual signal to be processed. (**c**) Top: analyzed propagation path. Bottom: final input sample (50 × 50 pixels, 1 channel modified color-map for better visualization).

Preliminary studies using 1D CNN yielded low performance in both training and testing phases. This motivated signal preprocessing to include both time and frequency domains in the same data input. The Morlet Wavelet Transform (MWT) was selected to convert the time-series signal into a time-frequency series, which allowed using them as inputs for a 2-D CNN. The Morlet complex wavelet was selected due to its suitability to extract features from UGW, as described in [29,30,31,32,33]. It is calculated through the convolution of the time-series signal with a wave-like oscillation called the mother wavelet, which is composed of a complex exponential carrier multiplied by a Gaussian window. The MWT was not applied directly to the acquired waveform. Instead, our algorithm worked with the residual signal obtained as the difference between the normalized responses to the 20-V and 10-V excitations, for the same conditions of frequency and toneburst cycles (this process reduced the available number of training samples by a factor of two, since each training sample needs for the signals from two tests). The residual signal of two responses at two different amplitudes of excitation was used to extract the nonlinearities inherent in the damage [46] by considering that the main source of nonlinearities of the system came from the damage itself [44]. In addition, the utilization of signals from the same structural state avoids the use of baseline data for future analysis and its consequent cost in data storage. The complete workflow is shown in Figure 7. Two different tests were carried out at two different levels of excitation (10 V and 20 V) with the same frequency and number of cycles of the toneburst. The signals were then normalized and the residual signal calculated and later transformed via MWT in a defined frequency range. Finally, the time-frequency 2-D matrix obtained from the wavelet transform was downsampled in the time domain to achieve the final size (in this study, it was set to 50 pixels corresponding to time domain).

The frequency-range analysis for the MWT was established to 50 equispaced intervals in the range of 100 kHz–600 kHz, and the time window was set to 0.16 ms duration (enough time to analyze the first arrival of the wave packets from S0 and A0 modes [47,48]). Moreover, final downsampling in the time-domain resulted in images of 50 × 50 pixels. This downsampling (reduced by a factor of 40) simplified implementing the data augmentation technique used to enhance the training. This point is explained in the following paragraphs.

### 3.4. Convolutional Neural Networks Design

**Figure 8:**
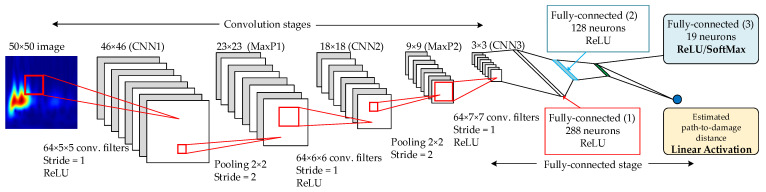
CNN proposed architecture. The final output layer depends on whether the CNN model is regression-based (linear activation, 1 neuron (yellow chart), previous layer with ReLU activation (blue chart) or classification-based (SoftMax activation in the last layer (blue chart)).

This section presents the architecture of two CNNs that have a common part and differ in the implementation of the output layer. One solution uses an output layer with only one neuron and a linear activation function (linear regression) that estimates the distance at where the impact is located. The other CNN includes an output layer with 19 outputs for classifying the discretized 18 interval distances or the pristine state using a SoftMax evaluation function. The architecture for both CNNs is depicted in Figure 8.

The designed CNN comprises two convolution layers with Max-Pooling layers stages, one Convolution layer, and three fully connected layers. The details, such as filter and pooling sizes and strides, are summarized in Table 2. In the case of regression-based CNN, the output is the path-to-damage normal distance, which makes it necessary to add an output neuron that calculates the linear regression from the previous layer. This regression-based CNN aims to directly estimate the target distance so that the data was arranged as 50 × 50 × N input training samples and an N × 1 numerical target array containing the calculated distances from the signal path to each training grid point. The input number of samples N is discussed in the following section.

**Figure 9:**
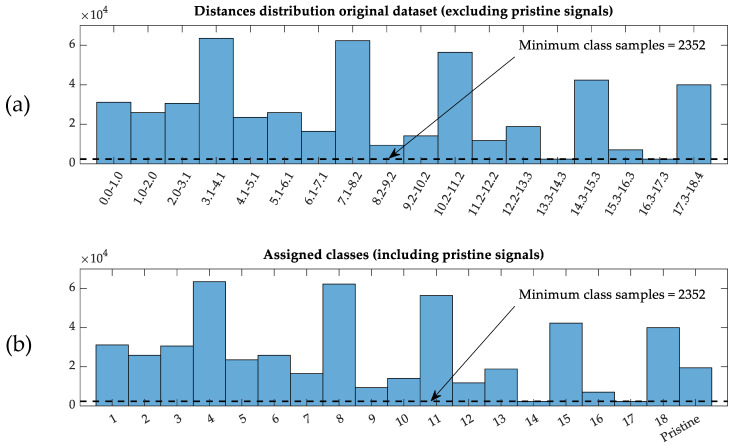
Histograms of the initial training dataset. (**a**) Discretized distribution of distances (in cm) of the original dataset (without those from the pristine state). (**b**) Distribution of classes for discretized distances and an additional class for pristine data.

The case of the classification-based CNN is slightly different from the regression-based one. The outputs are 19 different classes, divided as follows: the first 18 classes are the discretized target distance extracted from the dataset, in the same number of bins (data best fit is with this 18-bins distribution), and the last class corresponds to the pristine state, assigning every pristine signal to the same class label. This additional class allows considering the undamaged state in the model, which was not assigned to any distance. In the same way as the regression-based CNN model, the data was arranged as 50 × 50 × N input training samples, but the target array was considered as an N × 19 labels array, including the labeling for each distance (1, …, 18) and pristine (19) class (see Figure 9.).

The imbalanced distribution of the data among the histogram bins requires a further data arrangement, eliminating samples randomly to equilibrate the distribution (adapting to the minimum number of available samples), and thus avoiding bias in the training process.

#### 3.4.1. Improvement of the Training Dataset Using Data Augmentation

**Figure 10:**
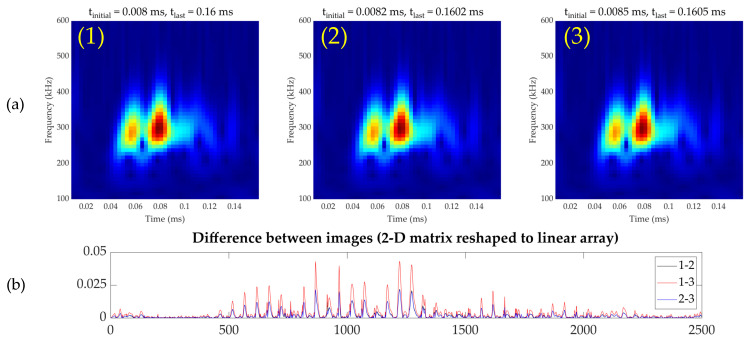
Data augmentation examples. (**a**) Different wavelet-transformed images obtained by moving the time window over the same signal. (**b**) Pixel-by-pixel difference between the images (1), (2), (3) in (**a**) in a linear array fashion.

To increase training samples for the network, data augmentation was carried out by slightly shifting the analysis window in Figure 7b and obtaining different images from the same time-series signal. The analysis window shift was set up to five time-domain samples, equivalent to a 0.4 μs of time delay in the maximum displacement case, resulting in a data augmentation of six times. Figure 10 shows examples of images obtained from the proposed augmentation and the difference among them.

**Figure 11:**
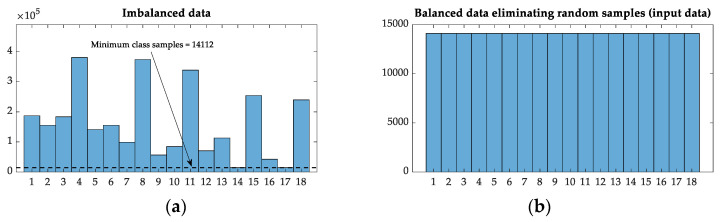
Histograms corresponding to the final training dataset. (**a**) Original dataset 6-times augmented, divided into 18 classes (pristine case excluded), showing the limiting number (which corresponds to classes 14 and 17). (**b**). Final input dataset, limited by the minimum number of samples per class from the augmented dataset.

As explained in the previous section, the original dataset, which was highly imbalanced concerning the number of samples per class, was reorganized to limit the number of samples per class. The reference value was obtained from the histogram distribution plot of the augmented dataset, which is the corresponding value to 14 and 17 classes (14,112 samples, Figure 11a). Finally, doing random selection before training, the final dataset was as shown in Figure 11b, with a uniform number of training samples per class, totaling 14,112 × 18 classes = 254,016 distance samples, and 14,112 pristine samples.

#### 3.4.2. Hyperparameters Tuning and Model Training

The training process was performed using Adaptative Moment Estimation (ADAM), a method for efficient stochastic optimization that only requires first-order gradients with fewer memory requirements [49]. The batch size was set to 2048 samples, and the number of training epochs was established at 500. Besides, some parameters were tuned to improve the training performance, such as a kernel regularization value of 0.0001 [50]; the kernel weights initializer was Glorot (also known as Xavier) [51].

#### 3.4.3. Training Software Tools

The software selected to develop the CNN models was based on TensorFlow tools. Anaconda (v.4.0) environment and Spyder IDE (v.4.2) with Python (v3.7) were chosen to ease the code development and debugging. TensorFlow-GPU backend (v.2.1.0) and Keras (v.2.2.4) were selected to design, analyze, and train the models. The training process was carried out using Nvidia Geforce RTX 2080. The computational training cost was around 1.5 h due to the use of the GPU in the training phase.

### 3.5. Distance Estimation-Based Imaging Algorithm

**Figure 12:**
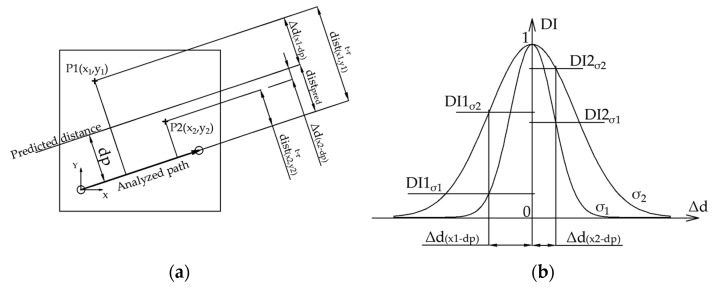
DI value assignment by the algorithm. (**a**) Distances layout for two different points, P1 (x_1_,y_1_) and P2 (x_2_,y_2_). P2 is more likely to be a damage point than P1 because it is closer to the predicted distance (the difference between actual distance and predicted distance is lower). Δd_i_ is the difference between predicted distance and pixel-path distance. (**b**) Designed Gaussian distribution, showing the corresponding DI values for the previous points represented by Δd_i_, for two different distributions with σ_2_ > σ_1_. Each distribution assigns a higher probability value to P2 because it is closer to the predicted distance.

With the main goal of obtaining an image of the damage index (between 0 and 1) for the plate, a specific algorithm was designed and implemented, receiving as input the estimated distances values obtained as outputs from the CNNs. In the case of the regression-based method, the distance output was directly fed to the imaging algorithm, but in the case of the classification-based method, each class was assigned to its corresponding numerical value. The algorithm was based on a geometric Gaussian distribution (see Equation (2)), assigning a higher value to the DI when the actual path-to-damage normal distance was closer to the predicted distance by the CNN, which means the analyzed point had a higher probability of being a damage location (Figure 12). Regarding Equation (2), the partial assigned value to *DI* is higher for the paths *t − r* for which the predicted distance is closer to the actual distance to the analysis point (*x,y*).

(2) $$DI\left(x,y\right)=\sum_{t=1}^{N}\sum_{r=1,\;\;r\neq t}^{N}\sum_{b=1}^{B}w_{b}\cdot e^{-\;\frac{{\left(dist_{\left(x,y\right)}^{\bar{t-r}}-dist_{pred\left(b\right)}\right)}^{2}}{2\sigma^{2}}},$$

where:

DI(x,y): damage index for the analyzed point.
*N*: number of attached transducers.
$dist_{\left(x,y\right)}^{\bar{t-r}}$: normal distance from the analysis point (*x,y*) to the line which intersects the transmitter and receiver transducers (*t* and *r*) path.
$dist_{pred\left(b\right)}$: predicted distance (corresponding distance-class value, in case of classification-based CNN) for the input signal (wavelet-transformed image).
σ: standard deviation of the exponential-Gaussian distribution. This value controls the influence of the propagation path on the analyzed point.
$w_{b}$: probability value for each *b* class in the t-r path signal prediction (only for classification-based CNN; otherwise, $w_{b}=1$). The value is obtained as output from the SoftMax layer of the model.
*B*: number of classes (only for classification-based CNN—18 distance classes, the pristine class has no associated distance value; otherwise, B=1).

## 4. Results

This section introduces the network training, validation, testing results, and imaging algorithm outcomes.

### 4.1. Training Process

**Figure 13:**
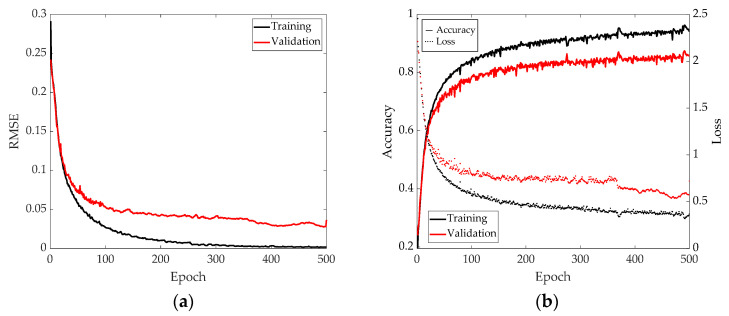
Training indicators evolution for (**a**) regression-based model (RMSE in cm) and (**b**) classification-based model; both accuracy (left vertical axis) and loss (right vertical axis).

The previous sections show how the training samples were distributed. The augmented dataset of 14,112 samples per class totaled 254,016 samples for the regression-based model (target value is the real number distance, not a class), and 14,112 × 19 = 268,128 samples for the classification-based model (adding the pristine class). The training, validation and testing dataset proportions were set to 70%, 25% and 5%, respectively, for each model’s training. Figure 13 shows the training process for both models, achieving 94.09% accuracy for training and 85.60% for validation in the classification-based model; the regression-based model had 0.203 cm error for training and 3.632 cm error for validation. The training process was carried out several times to check its stability, due to its stochastic nature.

Figure 13 shows two different results: for the regression-based model (Figure 13a). Its goodness of fit was evaluated through the RMSE (Root Mean Square Error) function, while for the classification-based model (Figure 13b), the goodness was evaluated through cross-entropy loss function, showing, in this case, both accuracy and loss values evolution.

### 4.2. Imaging Results

**Figure 14:**
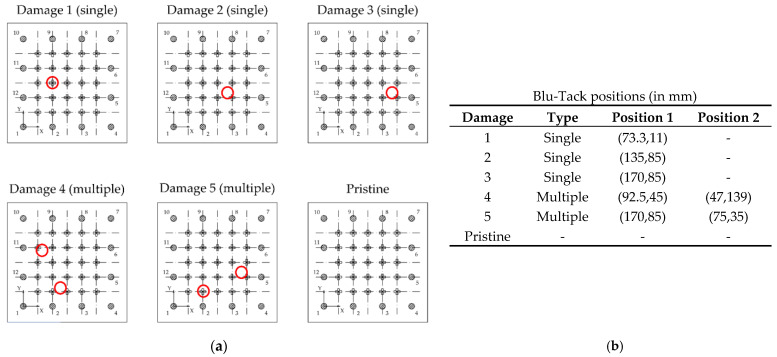
Additional tests for testing the method. (**a**) Simulated damage locations for testing the methodology. (**b**) Coordinates of the damage.

Several additional tests were carried out to check the effectiveness of the models, locating the Blu-Tack mass on other positions than previously trained: one slightly shifted from a training-grid position, two of them randomly located, two others by attaching two Blu-Tack masses at the same time, and finally, in a pristine state to evaluate the effectiveness of the additional pristine class in the classification-based model (Figure 14).

**Figure 15:**
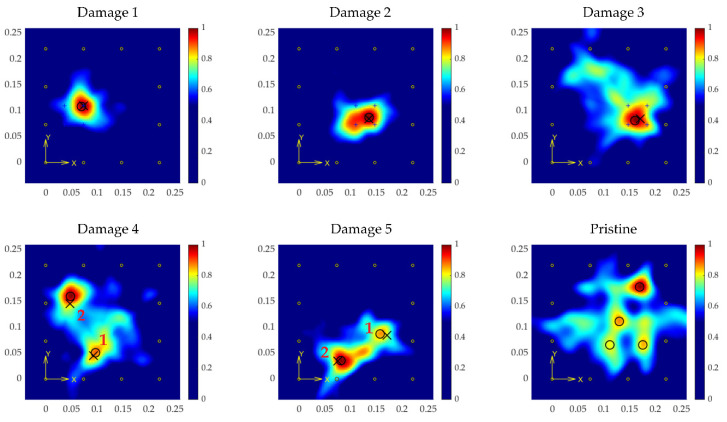
Results from the regression-based model for every test point (coordinates in m). Crosses point to the actual location of the damage, and circles to the estimated locations.

**Figure 16:**
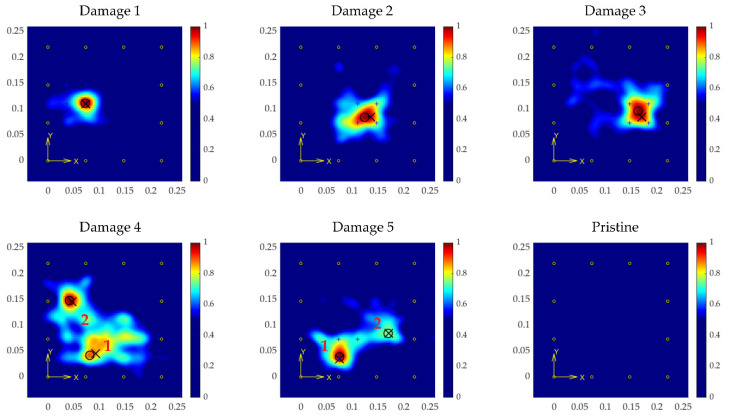
Results from the classification-based model for every test point (coordinates in m). Crosses point to the actual location of the damage, and circles to the estimated locations.

The results for tests, considering a grid size of 100 × 100 pixels (each pixel area is 9 mm^2^), and a σ value of 12.5 mm, are shown in Figure 15 for the regression-based model, and in Figure 16 for the classification-based model.

The images show high accuracy in the location, calculated in Table 3 and Table 4 (in terms of distance deviation from the predicted position to the actual location), even for the tests with multiple coexistent damage, a situation that was not trained for and expected by the model.

Of particular note is the pristine case. In the case of the regression-based model, the model estimated some distances inside the plate, resulting in the image shown in Figure 15 (Pristine), which shows several false positives. To avoid this effect, the added class in the classification-based model corresponding to pristine signals, and setting a threshold for the imaging process (in 80% of cases the signals are classified as pristine with the specimen undamaged), resulted in an all-zeroes surface, which means the analyzed structure is unaltered significantly (in this test the number of signals classified as pristine was 116/132 = 87.87%).

## 5. Discussion

Even though both models’ training processes showed an accuracy value of around 90%, the subsequent results, mainly achieved with the novel imaging algorithm, showed the full model (CNN plus imaging algorithm) as a reliable approach for accurate location of damage. Besides, the ability of the proposed method to detect multiple damage events, even though the training was carried out with single damage events, expands its possibilities in terms of generalization.

Blu-Tack simulated damage detection using the proposed CNN approaches resulted in accurate predictions for every additional test, as seen in Table 3 and Table 4. The maximum deviation obtained for any single simulated damage was less than 15 mm, and the mean value of the deviation for all single damage tests was approximately 5 mm, while the mean value for multiple damage was around 10 mm. Considering that the UGW S0 mode wavelength in the range of frequencies used in this study varied approximately between 0.02 m and 0.015 m, the obtained deviation values were within the range of Lamb waves sensitivity [43]. However, even though these results are promising, further validation on larger structures is needed.

The obtained map with the DI identifies the location of the individual damage without false positives, avoiding unnecessary additional inspections. Furthermore, the addition of a pristine class to the classification-based CNN helped to successfully evaluate the existence of damage (i.e., simple structure evaluation from the point of view of damaged-undamaged).

The presented imaging algorithm showed high performance for damage localization, even when multiple damage events were present at the same time. Its utilization instead of traditional imaging algorithms, such as RAPID [40] and Delay-and-Sum [52], showed better results when damage effects on UGW were slight, with the drawback of the previous training of the CNN for distance estimation.

## 6. Conclusions and Future Work

The work presented in this paper demonstrates the feasibility of 2D CNNs to estimate the damage index in a thermoplastic composite plate. The methodology followed to implement the solution using the Morlet wavelet transform and different CNN layers allow a solution generating an acceptable error in damage location. The ability of CNN for extracting damage-sensitive features from wavelet-transformed signals was shown with high effectiveness, with a special focus on normal path-to-damage distance, allowing triangulation of the position of the damage using the novel algorithm proposed for the methodology. The high accuracy of the prediction, obtained from the imaging process of the plate, makes the method a promising tool to be applied in SHM of aeronautical structures using PZT transducers.

The use of Blu-Tack for simulating damage (a well-established technique in UGW-based SHM research due to its scattering capabilities) should be changed by causing real damage, especially impact-induced delaminations, which are the most common damage sources in the aeronautical field when structures are manufactured with composite materials. Future research activity will include the execution of additional tests (in different environmental and operational conditions) in aeronautical facilities, applying the methodology developed in this work. The outcomes of these tests will allow improvement of the dataset to generate an enhanced CNN model, as well tuning the σ parameter in such a way that minimizes deviations in predictions with different transducer distributions.

Another consideration is the implementation of this methodology inside electronic embedded devices [45,53,54]. These devices enable internal data processing and real-time damage detection capabilities, making them very suitable for the SHM objective of autonomous structure evaluation. The application of software development and hardware acceleration tools, such as the OpenCL framework, will allow CNN model integration in autonomous embedded real-time systems.

## Figures and Tables

**Table 1:** Composite plate characteristics.

Matrix Polymer/Carbon Fiber	Layers	Ply/Total Thickness (mm)	Stacking Sequence
Tenax-E TPCL PEEK-HTA40/ Tenax-E HTA40 3K	9	0.31/2.79	[0/90/+45/ −45/0/90/+45/−45/0]

**Table 2:** CNN layers parameters.

Layer Type	Operation Size	Channels	Stride	Activation	Input Size	Output Size	Weights
Convolution (1)	5 × 5	64	1	ReLU	50 × 50 × 1	46 × 46 × 64	1664
Max-Pooling (1)	2 × 2	64	2	-	46 × 46 × 64	23 × 23 × 64	0
Convolution (2)	6 × 6	64	1	ReLU	23 × 23 × 64	18 × 18 × 64	147,520
Max-Pooling (2)	2 × 2	64	2	-	18 × 18 × 64	9 × 9 × 64	0
Convolution (3)	7 × 7	64	1	ReLU	9 × 9 × 64	3 × 3 × 64	200,768
Fully-Connected (1)	576 × 288	-	-	ReLU	3 × 3 × 64	288 × 1	166,176
Fully-Connected (2)	288 × 128	-	-	ReLU	288 × 1	128 × 1	36,992
Fully-Connected (3)	128 × 19	-	-	ReLU ^1^/SoftMax ^2^	128 × 1	19 × 1	2451
Fully-Connected (4)	19 × 1	-	-	Linear ^1^	19 × 1	1 × 1	20

^1^ Regression-based output layers (last one only in the regression-based model). ^2^ Classification-based output layer.

**Table 3:** Predicted locations and deviations of single damages (all units in mm).

		Regression-Based Model		Classification-Based Model	
Damage	Actual Location (mm)	Predicted Location (mm)	Deviation (mm)	Predicted Location (mm)	Deviation (mm)
1	(73.3,110)	(69.09,108.5)	4.47	(72.12,111.5)	1.92
2	(135,85)	(135.8,87.27)	2.39	(123.6,84.24)	11.39
3	(170,85)	(160,81.21)	10.69	(163,96.36)	13.33

**Table 4:** Predicted locations and deviations of multiple damages (all units in mm).

			Regression-Based Model				Classification-Based Model			
Dam.	Actual (1)	Actual (2)	Predicted (1)	Predicted (2)	Dev. (1)	Dev. (2)	Predicted (1)	Predicted (2)	Dev. (1)	Dev. (2)
4	(92.5,45)	(47,146)	(96.36,50.91)	(47.88,160)	7.06	14.02	(81.21,41.82)	(41.82,147.9)	11.72	5.51
5	(170,85)	(75,35)	(157,87.27)	(81.21,35.76)	13.22	6.25	(169.1,84.24)	(75.15,38.79)	1.18	3.79

## Data Availability

The data used in this study are available at https://github.com/i2a2/shm_cnn_datasets/sensors-21-05825 (accessed on 16 August 2021).
